# KRAS Mutations in Cancers: From Undruggable Dilemma to Precision Medicine

**DOI:** 10.1002/mco2.71050

**Published:** 2026-10-06

**Authors:** Jiaying Cai, Yimin Wu, Xing Jia, Li Yang, Wenjun Yang, Weishan Lu, Zhiye Huang, Yang Pan, Zixuan Fei, Junnan Ru, Jiazheng Zhao, Jue Shen, Taobo Luo, Da Chen, Haihong Yu, Haihan Deng, Jian Zeng, Wenmei Jiang, Leilei Wu

**Affiliations:** ^1^ Clinical Medical Academy Hangzhou Medical College (HMC) Hangzhou Zhejiang People's Republic of China; ^2^ Department of Anesthesiology Zhejiang Cancer Hospital Hangzhou Institute of Medicine (HIM) Chinese Academy of Sciences Hangzhou Zhejiang People's Republic of China; ^3^ Department of Thyroid Surgery Zhejiang Cancer Hospital Hangzhou Institute of Medicine (HIM) Chinese Academy of Sciences Hangzhou Zhejiang People's Republic of China; ^4^ Department of Gynecologic Oncology Zhejiang Cancer Hospital Hangzhou Institute of Medicine (HIM) Chinese Academy of Sciences Hangzhou Zhejiang People's Republic of China; ^5^ Department of Thoracic Surgery Zhejiang Cancer Hospital Hangzhou Institute of Medicine (HIM) Chinese Academy of Sciences Hangzhou Zhejiang People's Republic of China; ^6^ College of Traditional Chinese Medicine and Pharmacy Jinggangshan University Ji'an Jiangxi People's Republic of China

**Keywords:** acquired resistance, KRAS mutation, pan‐KRAS inhibitors, precision oncology, targeted therapy

## Abstract

KRAS is one of the most frequently altered oncogenic drivers across human solid tumors and has long represented a central challenge in precision oncology. The clinical success of covalent KRAS G12C inhibitors has transformed a historically “undruggable” target into a tractable therapeutic class, but durable benefit remains limited by adaptive feedback, acquired resistance, co‐mutation context, and tumor‐lineage‐specific barriers. A major unresolved question is how to extend this success across diverse KRAS alleles and cancer types. This review summarizes the molecular epidemiology and therapeutic landscape of KRAS‐mutant cancers, with emphasis on non‐small cell lung cancer, colorectal cancer, and pancreatic ductal adenocarcinoma. We compare G12C‐directed therapy with emerging G12D‐, G12V‐, and pan‐KRAS strategies and dissect resistance architectures involving EGFR feedback, RTK/MAPK/PI3K pathway reactivation, phenotypic plasticity, stromal constraints, and immune or metabolic adaptation. We further discuss resistance‐informed combination therapy, targeted protein degraders, immune‐based approaches, antibody–drug conjugates, biomarker‐guided sequencing, and strategies for rare KRAS variants. Particular attention is given to how allele identity, tumor lineage, and adaptive signaling jointly shape therapeutic vulnerability. By framing KRAS therapy through the “same mutation, different outcomes” paradigm, this review provides a context‐informed roadmap for extending KRAS‐directed precision oncology beyond G12C monotherapy.

## Graphical abstract

KRAS‐mutant cancers are therapeutically shaped by both allele identity and tumor context. Direct KRAS inhibition has achieved the greatest clinical success in KRAS G12C‐mutant NSCLC, whereas colorectal cancer often requires EGFR cotargeting and pancreatic cancer remains constrained by stromal and metabolic barriers. Shared resistance mechanisms include on‐target mutations, RTK bypass, MAPK/PI3K rebound, and tumor‐context constraints. Future advances will rely on next‐generation inhibitors, pan‐KRAS strategies, degraders, immune‐based approaches, and biomarker‐guided combination therapy.

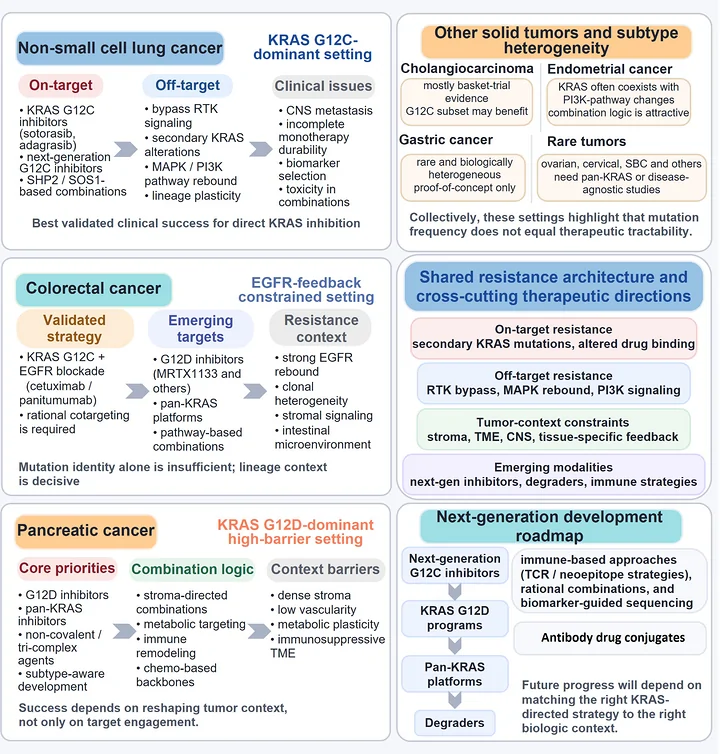

## 1 Introduction

Kirsten rat sarcoma viral oncogene homolog (KRAS) is one of the most frequently altered driver oncogenes across human malignancies [1]. The KRAS‐encoded small guanosine triphosphatase (GTPase) functions as a key molecular switch in the rat sarcoma viral oncogene homolog (RAS)–rapidly accelerated fibrosarcoma (RAF)–mitogen‐activated protein kinase kinase (MEK)–extracellular signal‐regulated kinase (ERK) signaling cascade, thereby regulating cell proliferation, differentiation, survival, and stress adaptation [2]. Under physiological conditions, KRAS dynamically cycles between an active guanosine triphosphate (GTP)‐bound state and an inactive guanosine diphosphate (GDP)‐bound conformation [3]. Oncogenic mutations disrupt the intrinsic GTP hydrolysis activity or attenuate their responsiveness to GTPase‐activating proteins, leading to persistent pathway activation and malignant progression [4]. Despite being identified more than four decades ago, KRAS remained a long‐standing “undruggable” target because of its relatively smooth protein surface and extremely high affinity for GTP, both of which limited the development of effective small‐molecule inhibitors [5, 6].

Importantly, KRAS‐mutant cancers do not represent a biologically uniform entity. The mutational spectrum of KRAS shows striking tumor‐type specificity, suggesting that the therapeutic implications of KRAS activation are profoundly shaped by tissue context. In lung cancer, particularly non‐small cell lung cancer (NSCLC), G12C is the dominant subtype and accounts for nearly 40% of KRAS alterations [7, 8, 9]. In contrast, G12D predominates in gastrointestinal malignancies such as pancreatic cancer and colorectal cancer (CRC), accounting for approximately 40%–50% and 30% of KRAS mutations, respectively [10, 11]. G12V is also commonly observed in pancreatic and colorectal cancers, whereas G13D is enriched in CRC and Q61H/L/R alterations are more frequently detected in melanoma and endometrial carcinoma [12]. These lineage‐dependent mutational patterns imply distinct evolutionary selection pressures, co‐mutation landscapes, and signaling dependencies across tumor types, underscoring the need for tumor‐context‐aware therapeutic strategies rather than a one‐size‐fits‐all view of KRAS‐directed treatment.

A major turning point was reached in 2021 with the approval of sotorasib (AMG‐510), the first covalent KRAS G12C inhibitor, which broke the long‐standing barrier to direct KRAS targeting. This advance was rapidly followed by the clinical development of additional KRAS G12C inhibitors, including adagrasib (MRTX849), which demonstrated meaningful antitumor activity in previously treated NSCLC [13, 14]. Mechanistically, these agents exploit the unique cysteine residue generated by the G12C substitution and irreversibly bind to the Switch‐II pocket, thereby stabilizing KRAS in its inactive GDP‐bound state [15, 16]. These achievements established proof of principle that direct KRAS inhibition is feasible in the clinic and reshaped the therapeutic landscape of KRAS‐mutant cancers.

However, the clinical success of KRAS‐targeted therapy remains highly context dependent. Although KRAS G12C inhibitors have produced clear benefit in NSCLC, their activity is substantially less impressive in other tumor types, particularly CRC [17]. In addition, many patients exhibit primary resistance, and most initial responders eventually develop acquired resistance, highlighting the limited durability of monotherapy [4, 18]. These findings indicate that the major challenge in KRAS‐directed therapy is no longer druggability alone, but whether durable pathway suppression can be achieved within a given tumor context. Co‐mutation patterns, adaptive receptor tyrosine kinase feedback, lineage plasticity, drug delivery barriers, central nervous system penetration, and tumor microenvironmental constraints all influence therapeutic response and resistance [19, 20, 21, 22]. Therefore, the same KRAS allele may carry markedly different biological and therapeutic consequences across different cancers.

In this review, we summarize recent advances and ongoing challenges in the treatment of KRAS‐mutant cancers from a tumor‐type‐specific perspective. We first outline the distribution and clinical relevance of major KRAS subtypes across distinct malignancies, with particular attention to G12C, G12D, G12V, G13D, and Q61 alterations. We then discuss the clinical development of direct KRAS inhibitors and emerging therapeutic modalities, including next‐generation inhibitors, degraders, and rational combination strategies. Importantly, we focus on why therapeutic outcomes differ across tumor types, dissecting both shared and context‐specific resistance mechanisms. The overall tumor‐type‐specific therapeutic logic, shared resistance architecture, and future development roadmap of KRAS‐directed therapy across major solid tumors are summarized in Figure 1. Through this framework, we aim to clarify how allele identity, tumor lineage, and adaptive signaling networks jointly determine therapeutic vulnerability, and to provide a more precise conceptual basis for future KRAS‐targeted treatment strategies.

**FIGURE 1:**
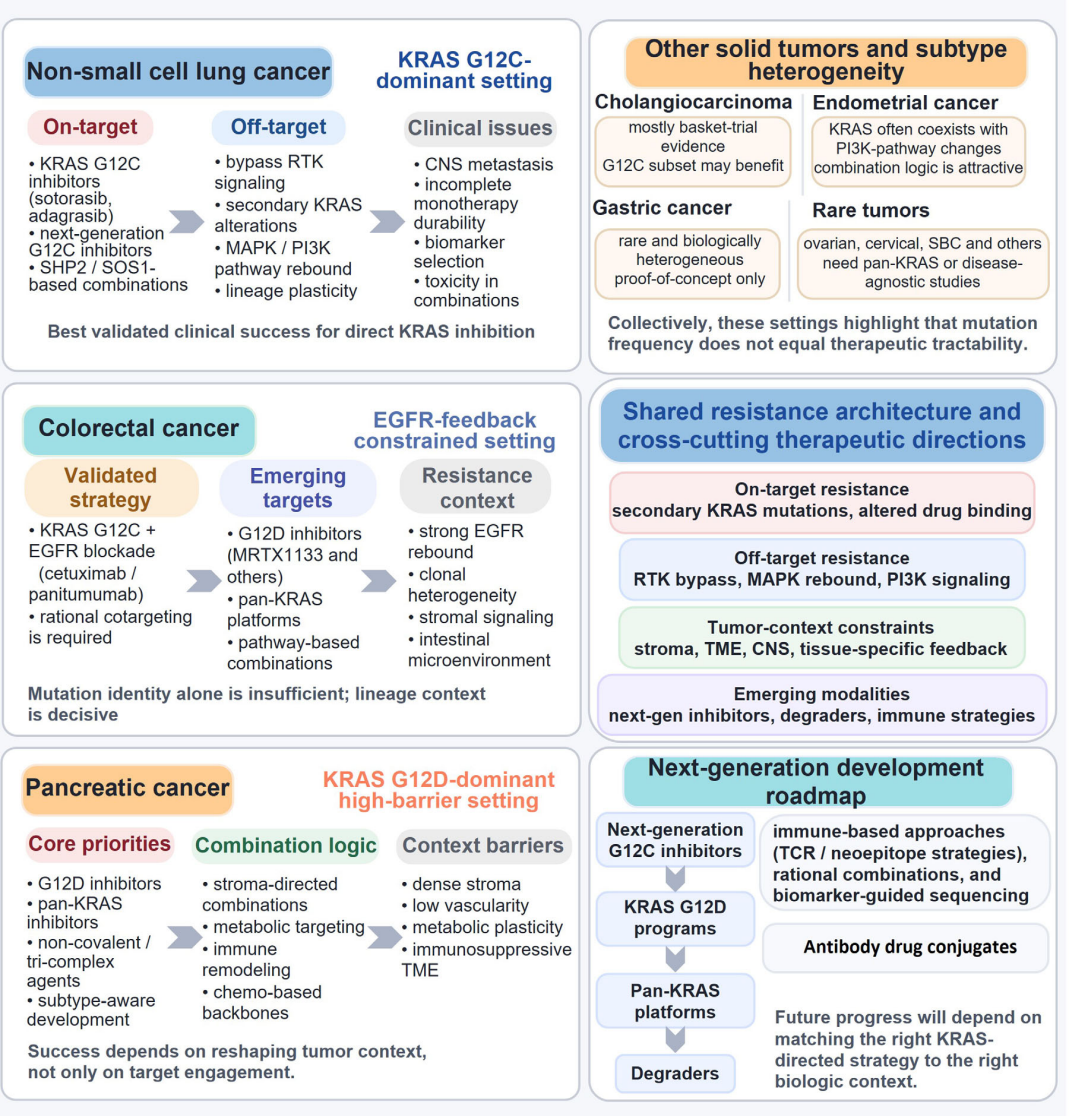
Tumor‐type‐specific therapeutic landscape and future directions of KRAS‐targeted therapy. The schematic summarizes the distinct therapeutic paradigms of KRAS‐mutant cancers across major solid tumors. In non‐small cell lung cancer (NSCLC), KRAS G12C represents the best‐validated setting for direct KRAS inhibition, although on‐target resistance, receptor tyrosine kinase (RTK) bypass signaling, downstream pathway rebound, central nervous system (CNS) metastases, and treatment‐related toxicity remain important challenges. In colorectal cancer (CRC), strong epidermal growth factor receptor (EGFR)‐mediated feedback limits KRAS G12C inhibitor monotherapy and supports EGFR‐based cotargeting, while G12D inhibitors and pan‐KRAS strategies are emerging. Pancreatic ductal adenocarcinoma (PDAC) is characterized by a G12D‐dominant mutation spectrum and substantial stromal, metabolic, and immunologic barriers, highlighting the need for subtype‐aware and tumor‐context‐directed combination strategies. Other solid tumors remain heterogeneous and are currently supported mainly by basket‐trial or early‐stage evidence. Across tumor types, shared resistance mechanisms include secondary KRAS alterations, RTK bypass activation, mitogen‐activated protein kinase (MAPK) and phosphoinositide 3‐kinase (PI3K) pathway rebound, and tumor‐context‐specific constraints. Future development is expected to integrate next‐generation G12C and G12D inhibitors, pan‐KRAS platforms, targeted protein degraders, immune‐based approaches, rational combinations, biomarker‐guided sequencing, and context‐directed modalities such as antibody‐drug conjugates. ADC, antibody‐drug conjugate; CNS, central nervous system; CRC, colorectal cancer; EGFR, epidermal growth factor receptor; MAPK, mitogen‐activated protein kinase; NSCLC, non‐small cell lung cancer; PDAC, pancreatic ductal adenocarcinoma; PI3K, phosphoinositide 3‐kinase; RTK, receptor tyrosine kinase; SBC, small bowel cancer; SHP2, Src homology phosphatase 2; SOS1, son of sevenless homolog 1; TCR, T‐cell receptor; TME, tumor microenvironment.

## 2 Non‐Small Cell Lung Cancer: Breakthroughs and Challenges in KRAS G12C‐Targeted Therapy

NSCLC represents the most clinically validated setting for direct KRAS inhibition, largely because of the relative prevalence and pharmacologic tractability of the G12C subtype. This section first reviews the structural basis and tumor distribution of KRAS G12C and summarizes the clinical development and current positioning of sotorasib, adagrasib, and other emerging inhibitors. It then examines the major limitations of current treatment, including incomplete monotherapy efficacy, primary and acquired resistance, central nervous system metastases, and treatment‐related toxicities. Finally, we discuss next‐generation G12C inhibitors, resistance‐informed upstream and downstream cotargeting, chemo‐/immunotherapy combinations, and emerging modalities such as KRAS degraders and immune‐based approaches. Collectively, NSCLC provides proof of concept for direct KRAS targeting while also illustrating the need to move beyond monotherapy toward deeper and more durable pathway control.

### 2.1 Molecular Features, Targeting Mechanism, and Cancer‐Type Distribution of KRAS G12C

The development of KRAS G12C‐targeted therapy was enabled by the discovery of an inducible allosteric pocket within the Switch‐II region of KRAS. In 2013, *Shokat* and colleagues showed that this pocket can be engaged by small molecules, thereby creating an opportunity for direct pharmacologic inhibition of mutant KRAS [21]. Structural studies subsequently demonstrated that ligand binding stabilizes the Switch‐II pocket and constrains KRAS in an inactive conformation, thereby suppressing nucleotide exchange and impairing downstream effector signaling [22, 23, 24]. A defining feature of this strategy is the covalent engagement of the mutant cysteine residue at codon 12, which confers allele selectivity and minimizes inhibition of wild‐type KRAS [25]. This mechanism formed the basis for the development of sotorasib and adagrasib, the first direct KRAS inhibitors to achieve clinical success in KRAS G12C‐mutant NSCLC [26, 27].

Importantly, the biological and therapeutic relevance of KRAS G12C is strongly shaped by tumor context. Among solid tumors, KRAS G12C is most prevalent in NSCLC, occurring in approximately 12%–14% of all NSCLC cases and accounting for up to 40% of KRAS‐mutant NSCLC [9, 28, 29]. By contrast, its frequency is much lower in CRC, at approximately 3%–4%, and even lower in pancreatic ductal adenocarcinoma (PDAC) and cholangiocarcinoma (CCA) [30, 31]. This lineage‐dependent distribution explains why NSCLC has become the principal setting for the clinical development of G12C inhibitors, whereas in lower‐frequency tumor types, particularly CRC, meaningful benefit has generally required biologically informed combination strategies rather than single‐agent therapy alone.

#### 2.1.1 Sotorasib and Adagrasib: Clinical Data and Current Clinical Positioning

Sotorasib was the first KRAS G12C inhibitor to demonstrate clinically meaningful activity in previously treated NSCLC. In phase II studies, sotorasib achieved an objective response rate (ORR) of approximately 35%–37%, a median progression‐free survival (PFS) of about 6–7 months, and a disease control rate (DCR) of around 80% in pretreated patients with KRAS G12C‐mutant NSCLC [19, 28, 32, 33]. Activity was also observed in selected patients with brain metastases, although the evidence base remains more limited than that for extracranial disease [34]. Treatment was generally manageable, with grade ≥3 adverse events occurring in a minority of patients and consisting mainly of gastrointestinal toxicity and liver function abnormalities [32, 34, 35].

The randomized phase III CodeBreaK 200 trial (NCT04303780) further established the clinical activity of sotorasib relative to docetaxel in previously treated advanced or metastatic KRAS G12C‐mutant NSCLC [36, 37]. In this study, sotorasib improved PFS and ORR compared with docetaxel, supporting its role as an active second‐line option. However, the magnitude of benefit remained modest, and no clear overall survival (OS) advantage was demonstrated, highlighting the limited durability of single‐agent KRAS G12C inhibition in this setting [36, 37]. Thus, although sotorasib represented a major therapeutic milestone, its clinical benefit also underscored the need for more effective sequencing strategies, biomarker refinement, and rational combination approaches.

Adagrasib has shown a broadly similar efficacy profile, with evidence of durable clinical activity in previously treated KRAS G12C‐mutant NSCLC. In clinical studies, adagrasib achieved an ORR of approximately 43%, a DCR of 80%, and a median duration of response (DOR) of more than 12 months in some cohorts [38]. Notably, adagrasib has generated encouraging intracranial data (KRYSTAL‐1, NCT03785249), particularly in patients with stable or selected untreated brain metastases, suggesting that central nervous system (CNS) activity may be achievable in appropriately selected patients [39]. Nevertheless, such findings should be interpreted cautiously, as CNS efficacy data remain more limited than systemic efficacy data.

In the phase III KRYSTAL‐12 trial (NCT04685135), adagrasib significantly improved PFS, ORR, and DOR compared with docetaxel in patients with previously treated advanced or metastatic KRAS G12C‐mutant NSCLC [40]. However, as with sotorasib, this benefit should not be overinterpreted as evidence of complete disease control, because acquired resistance remains common and the impact on OS requires cautious evaluation. Patients with active brain metastases were excluded, whereas those with stable brain metastases were eligible, and therefore statements regarding CNS activity should be restricted to these studied populations [40]. Overall, the currently available evidence supports adagrasib as an important treatment option after prior systemic therapy, while also emphasizing the need to move beyond monotherapy to achieve deeper and more durable responses.

Overall, sotorasib and adagrasib share broadly comparable toxicity profiles, with most treatment‐related adverse events being grade 1–2 and consisting mainly of diarrhea, nausea, vomiting, fatigue, anorexia, and transaminase elevation [35, 41]. Severe toxicities are less common but remain clinically relevant, particularly in the context of combination regimens. These agents have now been incorporated into treatment algorithms for previously treated KRAS G12C‐mutant NSCLC, but their ultimate value will depend on patient selection, resistance biology, and the development of more effective combination strategies [42].

#### 2.1.2 Other G12C Inhibitors and Upstream/Downstream Pathway Inhibitors

Beyond sotorasib and adagrasib, the therapeutic landscape of KRAS G12C inhibition is expanding rapidly [14]. A growing number of next‐generation KRAS G12C inhibitors have entered preclinical or early clinical development, with the aims of improving potency, CNS exposure, pharmacokinetics, and activity against resistance‐associated states or co‐alteration contexts [32, 38, 43, 44, 45, 46, 47]. Rather than ranking these agents across studies, it is more appropriate to view them as a developing class with heterogeneous clinical datasets, distinct trial designs, and currently limited head‐to‐head comparability. Figure 2 summarizes the chronological development of representative KRAS G12C inhibitors, whereas Table 1 provides an overview of representative clinical and preclinical therapeutic strategies targeting KRAS G12C and related signaling pathways.

**FIGURE 2:**
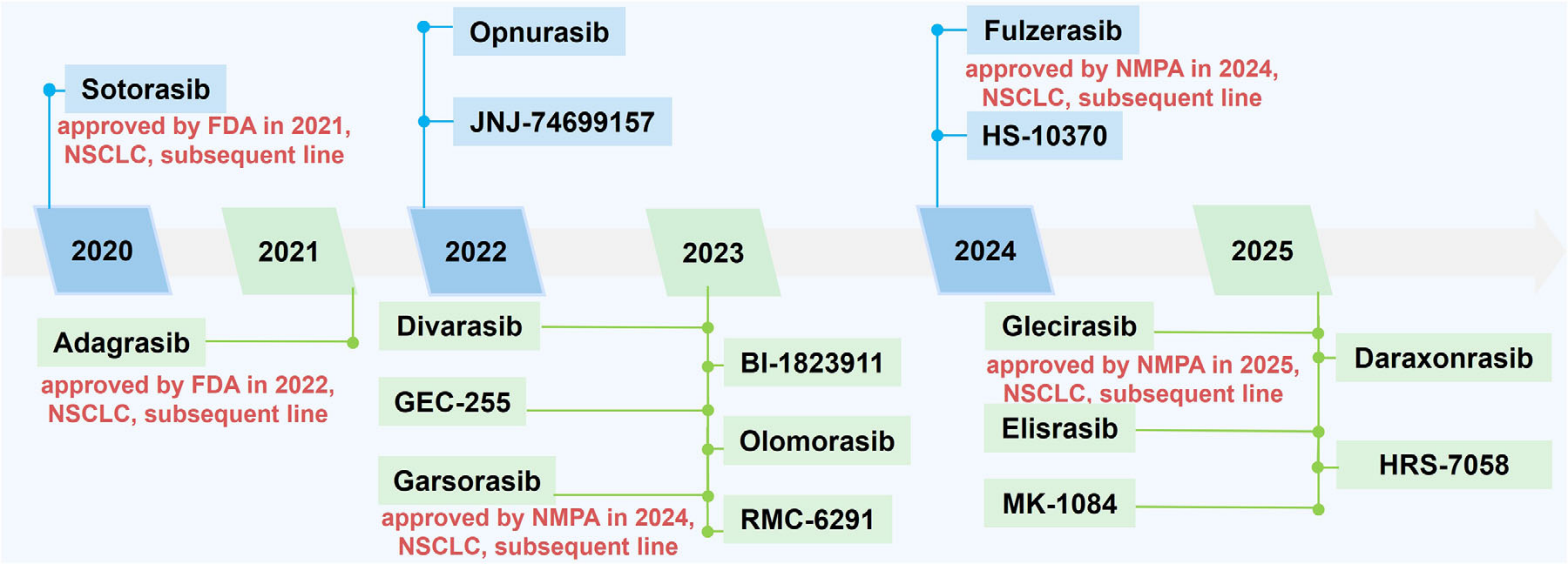
Timeline of representative KRAS G12C inhibitors and major clinical‐development milestones. Representative KRAS G12C inhibitors are arranged chronologically according to the year of their first publication or initial conference presentation. Regulatory milestones are highlighted in red, including approvals by the U.S. Food and Drug Administration (FDA) and the National Medical Products Administration (NMPA) for KRAS G12C‐mutant non‐small cell lung cancer (NSCLC). The timeline illustrates the rapid expansion of the KRAS G12C therapeutic landscape from the first clinically validated inhibitors to a growing pipeline of next‐generation agents under clinical development. FDA, U.S. Food and Drug Administration; NMPA, National Medical Products Administration; NSCLC, non‐small cell lung cancer.

**TABLE 1:** Representative KRAS G12C‐targeted and pathway‐based therapeutic strategies in solid tumors.

Therapeutic strategy	Trial (phase); treatment arms	Disease setting (*n*)	ORR (%)	Median PFS (months)	Median OS (months)	Refs.
**Direct KRAS G12C inhibitor**	CodeBreaK 100 (I/II); sotorasib 960 mg QD	After prior systemic therapy in NSCLC (≥1 line)	41	6.3	12.5	[34]
	CodeBreaK 200 (III); sotorasib vs. docetaxel	After platinum‐based chemotherapy and immunotherapy in NSCLC	28.1 vs. 13.2	5.6 vs. 4.5	10.6 vs. 11.3	[36]
	KRYSTAL‐1 (II); adagrasib 600 mg BID	NSCLC with untreated CNS metastases	42	5.4	11.4	[39]
	KRYSTAL‐1 (II); adagrasib 600 mg BID	KRAS G12C‐mutant solid tumors (excluding NSCLC and CRC)	35.1	7.4	NA	[48]
	KRYSTAL‐12 (III); adagrasib vs. docetaxel	After platinum‐based chemotherapy and anti‐PD‐1/PD‐L1 in NSCLC	32 vs. 9	5.5 vs. 3.8 (HR 0.58, 95% CI 0.45‐0.76)	NA	[40]
	Pivotal study (II); fulzerasib 600 mg BID	After failure of standard therapy (including brain metastases) in NSCLC (*n* = 116)	49.1	9.7	NA	[49]
	KontRASt‐01 (I/II); opnurasib vs. opnurasib + TNO155 (SHP2 inhibitor)	Advanced KRAS G12C‐mutant solid tumors	Early clinical antitumor activity observed	NA	NA	[44]
	NCT04956640 (I); olomorasib 50/ 100/ 150/ 200 mg QD	Advanced or metastatic solid tumors with KRAS G12C mutation	Non‐CRC, ORR: 37.4; CRC, ORR: 10.3	Non‐CRC median PFS: 6.9; CRC median PFS: 4.2	NE	[50]
**Direct KRAS G12C inhibitor + SHP2 inhibitor**	NCT05288205 (I/IIa); glecirasib (400/ 800 mg QD) + sitneprotafib (2/ 3 mg QD)	Locally advanced or metastatic KRAS G12C‐mutant NSCLC	Previously untreated, ORR: 71; pretreated, ORR: 49; pretreated with KRAS G12C Inhibitor, ORR: 10	Previously untreated, median PFS: 12.2; pretreated, median PFS: 10.8; pretreated with KRAS G12C Inhibitor, median PFS: 2.5	NA	[51]
	Preclinical; RMC‐4998 (RAS(ON) G12Ci) + SHP2 inhibitor	KRAS G12C‐mutant lung cancer (immune‐competent mouse models)	Suppressed tumor relapse, induced immune memory, sensitized to ICB	NA	NA	[52]
**Direct KRAS G12C inhibitor + EGFR inhibitor**	CodeBreaK 300 (III); sotorasib 960 mg + panitumumab vs. sotorasib 240 mg + panitumumab vs. investigator's choice (trifluridine/tipiracil or regorafenib)	Chemotherapy‑refractory KRAS G12C‐mutant metastatic CRC	30.2 vs. 7.5 vs. 1.9	5.7 vs. 4.01 vs. 2.04	NR vs. 11.9 vs. 10.3	[53]
	CodeBreaK 101 (Ib); sotorasib 480/ 720/ 960 mg + panitumumab	Chemotherapy‑refractory KRAS G12C‐mutant metastatic CRC	30	5.7	15.2	[54]
	KRYSTAL‐1 (II); adagrasib 600 mg BID vs. adagrasib 600 mg BID + cetuximab	Pretreated patients with metastatic CRC with mutant KRAS G12C	23 vs. 46	5.6 vs. 6.9	19.8 vs. 13.7	[55]
	JAB‐21822‐1002 and JAB‐21822‐1007 (I/II); glecirasib 800 mg QD vs. glecirasib 800 mg QD + cetuximab	Previously treated KRAS G12C‐mutant metastatic CRC	23 vs. 50	5.6 vs. 6.9	16.0 vs. 19.3	[56]
	NCT04449874 (Ib); divarasib 400 mg + cetuximab	Advanced or metastatic solid tumors with KRAS G12C mutation	62.5	8.1	NA	[57]
	KROCUS (I/II); fulzerasib 600 mg BID + cetuximab	KRAS G12C‐mutant NSCLC with no previous systemic anti‐tumor therapy for advanced or metastatic disease	69	12.5	NE	[58]
	NCT05410145 (I/II); elisrasib 50‐900 mg QD vs. elisrasib 600 mg QD + cetuximab	Locally advanced or metastatic KRAS G12C‐mutant CRC and PDAC	Elisrasib in CRC, ORR: 88.9; elisrasib in PDAC, ORR: 75.0; elisrasib + cetuximab in CRC or PDAC, ORR: NA	NA	NA	[59]
**Upstream/downstream pathway inhibitor**	NCT04252339 (I); Migoprotafib (SHP2 inhibitor), 10‐150 mg QD	Locally advanced or metastatic solid tumors (*n* = 56)	NA	NA	NA	[60]
	Preclinical; JAB‐3312 (sitneprotafib, SHP2 inhibitor)	Preclinical models	NA	NA	NA	[61]
	Preclinical; MRTX0902 (SOS1 inhibitor)	KRAS G12C‐mutant NSCLC and CRC models	NA	NA	NA	[62]
	Preclinical; HRX‐0233 (MAP2K4 inhibitor)	KRAS‐mutant lung and colon cancer cells	NA	NA	NA	[63]
	NCT06166836 / NCT05379946 (Ib/II); ifebemtinib 100 mg QD + garsorasib 600 mg BID	First‐line locally advanced or metastatic KRAS G12C‐mutant NSCLC (*n* = 33)	82	NA	NA	[64]
**Direct KRAS G12C inhibitor + Immunotherapy**	LOXO‐RAS‐20001 (I/II); olomorasib + pembrolizumab	Advanced KRAS G12C‐mutant NSCLC in any treatment line	57.1	NE	NA	[65]

Abbreviations: BID, twice daily; BRAF, B‐Raf proto‐oncogene; CI: confidence interval; CNS, central nervous system; CRC, colorectal cancer; EGFR, epidermal growth factor receptor; FAK, focal adhesion kinase; G12Ci, KRAS G12C inhibitor; HR, hazard ratio; ICB, immune checkpoint blockade; MEK, mitogen‐activated protein kinase kinase; MSI‐H, microsatellite instability‐high; mFOLFOX6, modified folinic acid, fluorouracil, and oxaliplatin; NA, not available; NE, not evaluated; NSCLC, non‐small cell lung cancer; OS, overall survival; ORR, objective response rate; PDAC, pancreatic ductal adenocarcinoma; PD‐L1, programmed death ligand 1; PD‐1, programmed cell death protein 1; PFS, progression‐free survival; pMMR, proficient mismatch repair' QD, once daily; SHP2, Src homology phosphatase 2; SOS1, son of sevenless homolog 1.

At the same time, KRAS‐directed therapy is increasingly being conceptualized beyond direct mutant blockade alone. Because pathway rebound and adaptive signaling can limit the activity of direct KRAS G12C inhibitors, multiple upstream and downstream nodes have emerged as rational cotargeting partners, including Src homology phosphatase 2 (SHP2), son of sevenless homolog 1 (SOS1), and phosphoinositide 3‐kinases (PI3K) [62, 66, 67, 68, 69]. These approaches are supported by a strong biological rationale, although their clinical activity has varied across different targets and therapeutic combinations [62, 70, 71, 72]. Accordingly, Figure 3 should be interpreted not simply as a pathway diagram, but as a framework for understanding why direct KRAS inhibition often requires network‐level therapeutic intervention.

**FIGURE 3:**
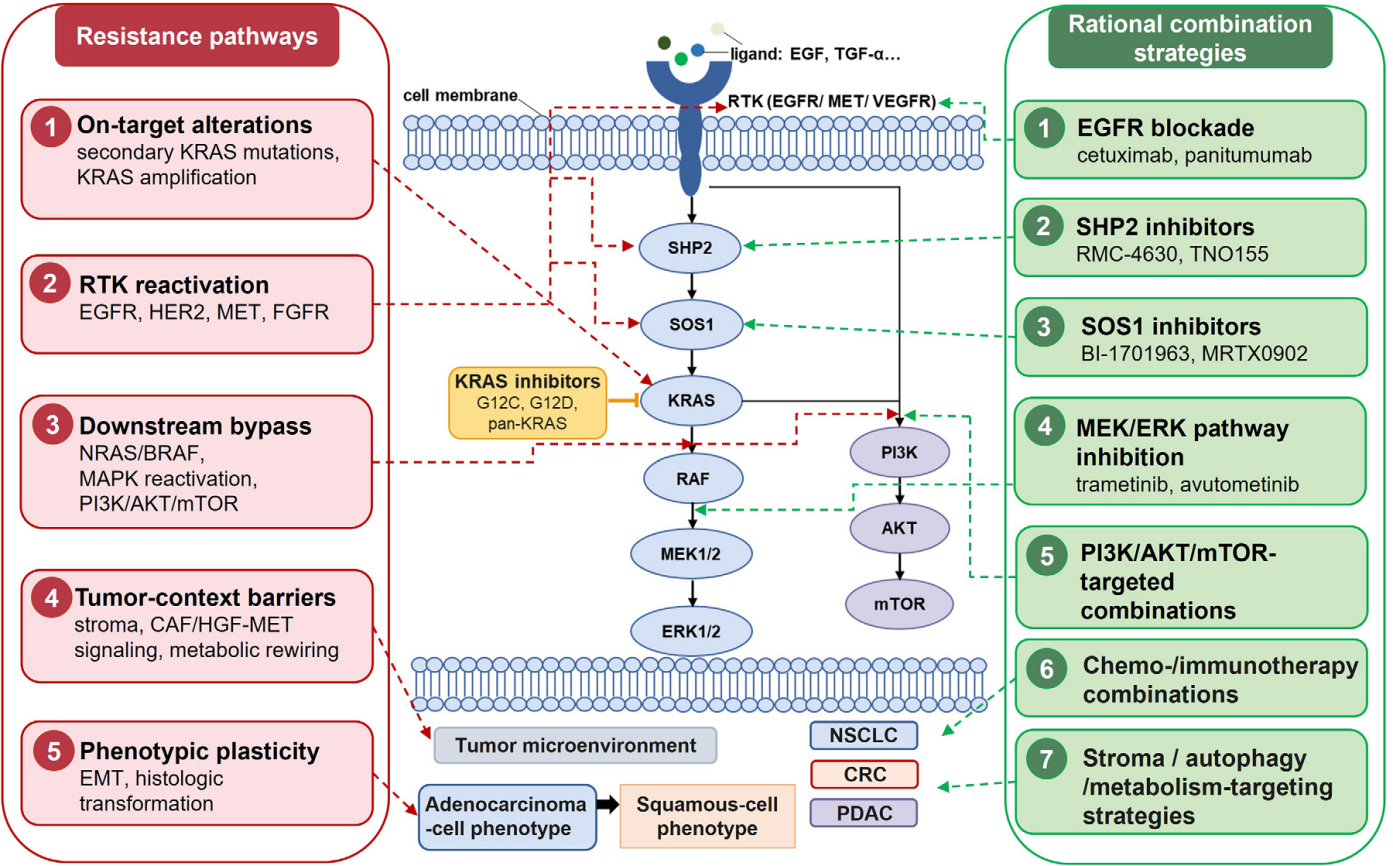
Major resistance mechanisms and rational combination strategies in KRAS‐targeted therapy. The schematic summarizes the major mechanisms of resistance to KRAS inhibition and the corresponding therapeutic strategies designed to suppress adaptive signaling and bypass pathways. Resistance mechanisms include on‐target alterations, such as secondary KRAS mutations and KRAS amplification; receptor tyrosine kinase (RTK) reactivation through EGFR, HER2, MET, or FGFR; downstream bypass signaling involving NRAS/BRAF, MAPK reactivation, and PI3K/AKT/mTOR signaling; tumor‐context‐dependent barriers, including stromal signaling, cancer‐associated fibroblast (CAF)/HGF–MET signaling, metabolic rewiring, and the tumor microenvironment; and phenotypic plasticity, including epithelial‐to‐mesenchymal transition (EMT) and histologic transformation from adenocarcinoma to squamous‐cell phenotypes. These mechanisms can restore or maintain oncogenic signaling despite direct inhibition of KRAS G12C, G12D, or other KRAS variants. Rational combination strategies include EGFR blockade, SHP2 and SOS1 inhibition, MEK/ERK pathway inhibition, PI3K/AKT/mTOR‐targeted therapy, chemo‐/immunotherapy combinations, and stroma‐, autophagy‐, or metabolism‐directed approaches. Solid black arrows indicate canonical signal transduction, red dashed arrows indicate representative resistance or pathway‐reactivation routes, green dashed arrows indicate therapeutic intervention points or context‐specific combination strategies, and the inhibitory bar indicates direct KRAS blockade. The lower panel further illustrates the contribution of the tumor microenvironment and phenotypic plasticity to tumor‐type‐specific resistance patterns. AKT, protein kinase B; CAF, cancer‐associated fibroblast; CRC, colorectal cancer; EGF, epidermal growth factor; EGFR, epidermal growth factor receptor; EMT, epithelial‐to‐mesenchymal transition; ERK, extracellular signal‐regulated kinase; FGFR, fibroblast growth factor receptor; HER2, human epidermal growth factor receptor 2; HGF, hepatocyte growth factor; MAPK, mitogen‐activated protein kinase; MEK, mitogen‐activated protein kinase kinase; MET, MET proto‐oncogene receptor tyrosine kinase; mTOR, mechanistic target of rapamycin; NSCLC, non‐small cell lung cancer; PDAC, pancreatic ductal adenocarcinoma; PI3K, phosphoinositide 3‐kinase; RAF, rapidly accelerated fibrosarcoma; RTK, receptor tyrosine kinase; SHP2, Src homology phosphatase 2; SOS1, son of sevenless homolog 1; TGF‐α, transforming growth factor alpha; VEGFR, vascular endothelial growth factor receptor.

### 2.2 The Core Challenges of Current Treatment

#### 2.2.1 Limited Efficacy and Acquired Resistance

A central limitation of first‐generation KRAS G12C inhibitors is that, despite their clear clinical activity, their efficacy as monotherapy remains incomplete and highly context dependent. In NSCLC, ORRs with sotorasib and adagrasib are generally in the 30%–40% range, whereas in CRC the corresponding single‐agent response rates are markedly lower, typically around 10%–20% [19, 28, 32, 33]. These observations indicate that allele‐specific druggability alone is insufficient to ensure durable pathway suppression, and that therapeutic efficacy is strongly modulated by co‐mutation patterns, receptor tyrosine kinase (RTK) feedback, tissue‐specific drug exposure, and the tumor microenvironment [4, 41, 73].

Mechanistically, first‐generation covalent KRAS G12C inhibitors preferentially bind the inactive, GDP‐bound state of KRAS G12C, but do not fully suppress the active, GTP‐bound pool. This creates an inherent vulnerability to pathway reactivation, particularly through upstream RTK signaling and downstream signaling bypass. As a result, the depth and durability of response are limited even in initially sensitive tumors, and these limitations are further amplified by concurrent genomic alterations such as KRAS amplification or RTK pathway co‐mutations. In this sense, the major challenge of KRAS G12C therapy is not simply resistance after response, but incomplete pathway control from the outset.

Acquired resistance has emerged as a near‐universal challenge in patients who initially benefit from KRAS G12C inhibitors. Clinical progression typically occurs within months, reflecting the selective pressure imposed by treatment and the evolutionary plasticity of KRAS‐driven tumors [73]. On‐target resistance includes secondary KRAS alterations that impair drug binding or restore signaling, such as substitutions affecting codons 12, 61, 95, or 96 [41]. In response, multiple strategies are being explored, including next‐generation KRAS inhibitors capable of overcoming specific resistance mutations, as well as combination approaches targeting proximal signaling mediators such as SHP2/SOS1 or distal MAPK effectors such as MEK (Table 1) [73].

Off‐target resistance is equally important and includes bypass pathway activation, downstream pathway substitutions, and parallel pathway reactivation (summarized in Figure 3). Representative mechanisms include EGFR or MET amplification, neuroblastoma RAS viral oncogene homolog (NRAS) or BRAF alterations, and re‐engagement of PI3K/ protein kinase B (AKT)/ mechanistic target of rapamycin (mTOR). These mechanisms highlight that resistance to KRAS G12C inhibition is not monolithic, but instead reflects multiple layers of signaling redundancy and lineage‐specific adaptation. Therefore, combination therapy should be guided by the dominant resistance architecture in each disease context rather than applied uniformly across tumor types.

Phenotypic plasticity provides an additional layer of resistance complexity. Histologic or transcriptional state changes, including adenosquamous transformation (Figure 3), have been implicated in resistance to KRAS‐directed therapy in lung cancer [74]. In such cases, the tumor may retain KRAS G12C while becoming less dependent on the signaling context in which the inhibitor was originally effective. These observations reinforce the idea that resistance is not only a molecular problem of altered drug binding, but also a biological problem of lineage reprogramming, altered cell state, and adaptive survival signaling.

#### 2.2.2 CNS Metastasis and Treatment‐Related Toxicity

CNS metastasis remains a major unmet need in advanced KRAS G12C‐mutant NSCLC. Brain metastases are common in this population and contribute substantially to morbidity and mortality. However, the efficacy of first‐generation KRAS G12C inhibitors in the CNS is constrained by limited blood‐brain barrier penetration, active efflux transport, and the relative scarcity of prospective intracranial efficacy data [75, 76]. Although adagrasib has shown encouraging intracranial activity in selected patients, especially in the KRYSTAL‐1 dataset, CNS control remains less robust than extracranial control, and further optimization of CNS‐active KRAS inhibitors remains a clear priority.

Another major challenge is treatment‐related toxicity, particularly in the setting of combination therapy. While single‐agent KRAS G12C inhibitors are generally manageable, combination strategies with immunotherapy, chemotherapy, or other targeted agents may increase treatment‐related toxicity and complicate dose intensity, scheduling, and treatment adherence [77, 78]. In particular, hepatotoxicity, gastrointestinal toxicity, and pulmonary toxicity have emerged as recurrent concerns in early combination studies. Accordingly, the next phase of clinical development should not focus on efficacy alone, but also on how to optimize sequencing, dose modification, and combination design in order to preserve tolerability while improving antitumor activity.

### 2.3 Frontier Research Advances and Therapeutic Strategy Exploration

#### 2.3.1 Next‐Generation Development of KRAS G12C Inhibitors

The next wave of KRAS‐directed drug development is focused on three broad goals: improving the potency and pharmacologic properties of direct KRAS G12C inhibitors, extending coverage across multiple KRAS alleles, and developing new modalities capable of overcoming resistance‐associated conformational states. In this context, next‐generation KRAS G12C inhibitors are being evaluated for enhanced target engagement, improved CNS exposure, and greater activity in tumors with intrinsic or acquired resistance to first‐generation compounds [79]. These agents may help address current limitations of single‐agent G12C inhibition, but their ultimate value will depend on whether improved biochemical properties translate into superior clinical durability.

Pan‐KRAS inhibitors represent a conceptually attractive strategy because they may cover multiple mutant alleles, including G12C, G12D, G12V, and other recurrent variants (Table 2). Such agents could be particularly valuable in tumors with heterogeneous KRAS mutation patterns or in settings where allele‐specific inhibitors are not yet clinically available. However, pan‐KRAS development also raises important translational challenges, including the therapeutic window, mutant selectivity, and the balance between broader pathway suppression and acceptable toxicity [80]. Thus, although pan‐KRAS inhibition is an exciting frontier, it should still be regarded as an evolving strategy rather than a solved therapeutic solution.

**TABLE 2:** Representative pan‐KRAS inhibitors and therapeutic strategies for uncommon KRAS variants in preclinical and clinical development.

Therapeutic strategy	Trial (phase); treatment arms	Disease setting (*n*)	ORR (%)	Median PFS (months)	Median OS (months)	Refs.
**Pan‐KRAS inhibitor (monotherapy)**	NCT05379985 (I/II); daraxonrasib (RMC‐6236) 300 mg QD	Second‐line RAS G12‐mutant advanced PDAC (*n* = 26)	35	8.5	13.1	[81]
	NCT05379985 (I/II); daraxonrasib (RMC‐6236)	Previously treated advanced RAS‐mutant NSCLC (*n* = 136)	300 mg QD: 37	NA	NA	[82]
	NCT06625320 (III); Daraxonrasib vs. chemotherapy	Metastatic PDAC with RAS mutations	daraxonrasib: 31.6; chemotherapy: 11.2	daraxonrasib: 7.2; chemotherapy: 3.6; HR = 0.49	daraxonrasib: 13.2; chemotherapy: 6.7; HR = 0.4	[83]
**Pan‐KRAS inhibitor + KRAS G12C inhibitor (doublet)**	M23RMC(Ib); RMC‐6236 + RMC‐6291	Advanced KRAS G12C‐mutant solid tumors (including NSCLC with prior G12Ci)	Recruiting	NA	NA	[13]
**KRAS G12V inhibitor (RNA therapy)**	Preclinical (IND‐track); EFTX‐G12V	KRAS G12V‐mutant tumors (lung, CRC, pancreatic)	Significant tumor growth inhibition and CRs in mouse models	NA	NA	[84]
**KRAS G12D/V siRNA**	Phase II (NCT01676259); siG12D‐LODER + chemotherapy	Cohort 1 (G12D/V): *n* = 16 (11 in experimental arm, 5 in control) Cohort 2 (G12D/V): *n* = 14 (single arm)	Cohort 2 (G12D/V), ORR: 57.1; Cohort 1, ORR: 63.6; Cohort 2 (overall), ORR: 31.6	NA	G12D/V subgroup (Cohort 1): 22.7 (siG12D‐LODER + chemotherapy) vs. 13.5 (chemotherapy) (HR = 0.59)	[85]
**MEK inhibitor + pan‐RTK inhibitor**	NCT04967079(Ia); trametinib + anlotinib	Advanced non‐G12C KRAS‐mutant NSCLC (*n* = 13)	69.2	6.9	NA	[86]
	NCT04967079(Ib); trametinib + anlotinib	Advanced non‐G12C KRAS‐mutant NSCLC (n=20)	65	11.5	15.5	[86]
**PanRAF inhibitor + SRC inhibitor**	NCT02437227(I); CCT3833	Multiple KRAS‐mutant solid tumors (including G12V sarcoma case report)	Preclinical efficacy; exceptional PFS in G12V sarcoma case	NA	NA	[87]

Abbreviations: 2L, second‐line; 3L, third‐line; CRC, colorectal cancer; CR, complete response; G12Ci, KRAS G12C inhibitor; HR, hazard ratio; IND, investigational new drug; MEKi, MEK inhibitor; NA, not available; NE, not evaluated; NSCLC, non‐small cell lung cancer; ORR, objective response rate; PDAC, pancreatic ductal adenocarcinoma; PFS, progression‐free survival; OS, overall survival; QD, once daily; RNA, ribonucleic acid; RTKi, receptor tyrosine kinase inhibitor; siRNA, small interfering RNA; SRC, SRC proto‐oncogene tyrosine‐protein kinase.

Additional innovation is emerging from non‐covalent and multivalent inhibitor platforms. Unlike first‐generation covalent KRAS G12C inhibitors, non‐covalent inhibitors do not rely on irreversible engagement of Cys12 and may therefore retain activity in certain resistance settings [88]. Tri‐complex RAS(ON) inhibitors, by simultaneously engaging more than one functional surface of KRAS, may further enhance binding affinity and suppress signaling more effectively than single‐site inhibitors [89]. Together, these approaches broaden the conceptual landscape of KRAS targeting beyond the initial G12C paradigm and may help address both resistance and allele diversity in future therapeutic development.

#### 2.3.2 Combination Therapy: A Key Path to Overcoming Drug Resistance

Combination therapy is increasingly viewed as the most plausible route to deeper and more durable control of KRAS‐mutant tumors. The rationale is straightforward: direct KRAS inhibition can suppress oncogenic signaling, but adaptive feedback, pathway redundancy, and tumor microenvironmental constraints often permit residual disease to persist. Rational combinations aim to block these escape routes and thereby increase both the depth and durability of response.

One important area of interest is the combination of KRAS G12C inhibitors with immunotherapy. Preclinical and translational studies suggest that KRAS inhibition can partially remodel the tumor microenvironment, attenuate immunosuppressive signaling, and potentially increase sensitivity to PD‐1/PD‐L1 blockade. Early clinical studies, including trials of olomorasib plus pembrolizumab, have reported encouraging activity and manageable safety in selected populations (Table 1), particularly in treatment‐naive patients and in those with higher PD‐L1 expression [65]. Beyond direct KRAS inhibitor–ICI combinations, co‐mutation‐defined immune states may provide additional therapeutic opportunities. In KRAS–threonine kinase 11 (STK11/LKB1) co‐mutant NSCLC, targeted phosphoglycerate mutase family member 5 (PGAM5) silencing combined with decitabine activated the cyclic guanosine monophosphate (GMP)–adenosine monophosphate (AMP) synthase (cGAS)–stimulator of interferon genes (STING) signaling and remodeled the immunologically “cold” tumor microenvironment, thereby enhancing the antitumor efficacy of PD‐1 blockade [90]. Nevertheless, these findings remain preliminary, and the trade‐off between improved efficacy and increased toxicity requires careful prospective evaluation.

Combination strategies with chemotherapy also merit attention. Compared with immunotherapy‐containing regimens, chemotherapy‐based combinations may provide more immediate cytoreduction and may be particularly relevant in patients with high disease burden or rapidly progressive disease [91, 92, 93]. However, cross‐trial comparisons remain inherently limited, and it is premature to conclude that any one combination platform is definitively superior. At present, the key message is that the future of KRAS G12C therapy is likely to depend on context‐matched combination design rather than on indefinite optimization of monotherapy alone.

#### 2.3.3 Innovative Drug Modalities and Technologies

Beyond small‐molecule inhibition, several innovative therapeutic modalities are being developed to target KRAS more directly or more selectively. Among these, proteolysis‐targeting chimeras (PROTACs) have attracted considerable interest. Since 2020–2021, multiple KRAS G12C‐directed degraders, including LC‐2 and reversible covalent PROTAC series, have been reported to induce substantial degradation of KRAS G12C protein in vitro [94, 95]. By promoting ubiquitination and subsequent proteasomal degradation, these agents may circumvent some of the limitations associated with occupancy‐driven inhibition alone.

Cellular immunotherapy is also emerging as a potentially transformative approach. HLA‐restricted T‐cell receptor (TCR)‐based strategies targeting recurrent KRAS mutations are being developed for adoptive cellular therapy. Clinical proof of concept has been demonstrated for KRAS G12D‐ and G12V‐specific TCR‐T approaches in selected patients [96, 97, 98, 99]. In parallel, recent studies suggest that drug‐bound KRAS neoepitopes may be presented by major histocompatibility complex class I molecules, opening the possibility of combining targeted therapy with bispecific immune engagement or engineered T‐cell approaches [97]. Although these strategies remain early in development, they illustrate how KRAS targeting is moving beyond kinase‐like pharmacology toward multi‐platform precision oncology.

## 3 Colorectal Cancer: From G12C Combination Breakthroughs to G12D Targeting Explorations

CRC provides a paradigmatic example of how the same KRAS mutation can produce different therapeutic outcomes across tumor lineages. This section first outlines the heterogeneous distribution and clinical relevance of KRAS subtypes in CRC. It then explains why the limited single‐agent activity of KRAS G12C inhibitors in CRC is largely driven by adaptive epidermal growth factor receptor (EGFR) feedback and why simultaneous EGFR blockade has become the best‐validated therapeutic strategy in this setting. The emerging development of G12D inhibitors is subsequently reviewed, together with the unresolved therapeutic significance of G13D, G12V, Q61, and other less tractable RAS variants. Finally, we examine CRC‐specific resistance determinants, including EGFR‐dependent pathway reactivation, clonal heterogeneity, stromal signaling, and intestinal microenvironmental influences. These features underscore that allele identity alone is insufficient and that successful KRAS‐directed therapy in CRC requires both subtype‐specific and lineage‐adapted treatment design.

### 3.1 Molecular Epidemiology of KRAS Mutations in Colorectal Cancer

KRAS is one of the most frequently mutant driver genes in CRC, occurring in approximately 35%–45% of cases and serving as a well‐established determinant of resistance to anti‐EGFR monoclonal antibodies such as cetuximab and panitumumab [100, 101]. For many years, this made KRAS‐mutant CRC a prototypical example of a molecularly defined but therapeutically underserved disease. The recent emergence of direct KRAS‐targeted strategies, particularly for G12C, has begun to change this paradigm [102].

The subtype distribution of KRAS mutations in CRC is highly heterogeneous and has important therapeutic implications. G12D is the most common subtype, accounting for approximately 30%–40% of KRAS mutations, followed by G12V and G13D [103, 104, 105]. By contrast, G12C is relatively uncommon, representing only a small subset of CRC, but its unique cysteine residue makes it the first KRAS subtype in CRC to become clinically targetable [19]. This distribution is clinically meaningful because it means that the first successful wave of KRAS‐targeted therapy in CRC has emerged not from the most common allele, but from the most tractable one.

The therapeutic implications of these subtypes are clearly distinct. G12C has become the leading example of successful biologically informed cotargeting in CRC, whereas G12D remains the major unmet need because of its higher prevalence and more challenging chemistry [88, 106]. G12V and other rare subtypes currently lack mature allele‐specific treatment options, and their management is more likely to depend on pan‐KRAS strategies or broader pathway‐based approaches [107, 108]. Thus, subtype heterogeneity is not merely a molecular classification issue in CRC; it is a central determinant of therapeutic strategy and clinical trial design.

### 3.2 G12C Mutation in CRC: Overcoming EGFR‐Mediated Resistance With Combination Therapy

CRC provides one of the clearest examples that the presence of a targetable KRAS allele does not guarantee equivalent benefit across tumor types. Unlike in NSCLC, where KRAS G12C inhibitors have meaningful single‐agent activity, monotherapy in CRC has produced substantially lower response rates [32]. The principal reason is adaptive EGFR‐mediated feedback activation: when KRAS G12C is inhibited in CRC cells, upstream RTK signaling, particularly EGFR, is rapidly re‐engaged, restoring MAPK pathway output and limiting the depth and duration of response [18]. This mechanism is more prominent in CRC than in NSCLC and reflects the lineage‐specific signaling dependence of colorectal epithelium.

This biological insight has directly shaped clinical strategy. Combining KRAS G12C inhibitors with EGFR monoclonal antibodies has emerged as the most successful approach to date for KRAS G12C‐mutant CRC (Table 1). In CodeBreaK 101, sotorasib plus panitumumab improved response and disease control relative to historical expectations for monotherapy [19, 109]. Similarly, adagrasib plus cetuximab has shown encouraging activity, and this dual‐targeting concept has now moved beyond proof‐of‐concept toward regulatory and clinical implementation in previously treated metastatic KRAS G12C‐mutant CRC [19, 110]. These data support a broader principle: in CRC, the key question is often not whether KRAS can be inhibited, but whether compensatory EGFR signaling is simultaneously suppressed. Other combination strategies, including cotargeting of SHP2 or incorporation of chemotherapy, are also being explored [52]. However, the EGFR cotargeting model currently represents the most convincing example of tumor‐type‐specific therapeutic adaptation in KRAS‐mutant CRC and illustrates how biologic context can reshape the clinical utility of a given KRAS‐directed drug.

### 3.3 G12D Mutation: A New Frontier in Targeted Therapy

#### 3.3.1 Technical Challenges and Development Progress in Targeting G12D

KRAS G12D is the most common KRAS mutation subtype in CRC, but it has historically been far more difficult to target than G12C. The major challenge arises from the biochemical properties of the aspartic acid substitution: unlike G12C, G12D does not provide a cysteine residue for covalent drug engagement, forcing drug developers to rely on highly optimized non‐covalent binding strategies [106]. As a result, KRAS G12D has long been regarded as one of the most important yet technically challenging targets in precision oncology. Given its prevalence in CRC, however, effective G12D inhibition remains a major unmet clinical need.

Recent years have brought meaningful progress in this area. MRTX1133 was the first highly potent and selective non‐covalent KRAS G12D inhibitor to demonstrate robust preclinical antitumor activity. In KRAS G12D‐mutant CRC models, MRTX1133 suppresses oncogenic signaling, although adaptive EGFR–wild‐type RAS feedback can limit its efficacy and provides a rationale for cotargeting EGFR [88, 111, 112]. Additional agents, such as HRS‐4642 and RMC‐9805, have further expanded the early G12D development landscape, while other candidates remain in preclinical optimization [88, 106]. Importantly, MRTX1133 has entered early clinical development, underscoring that G12D targeting has moved from a purely conceptual objective into a translational reality, although its ultimate clinical role remains to be defined. Table 3 highlights representative agents that remain in preclinical or early‐phase evaluation.

**TABLE 3:** Representative KRAS G12D‐targeted therapeutic strategies in preclinical and early clinical development.

Therapeutic strategy	Trial (phase); treatment arms	Disease setting	ORR (%)	Median PFS (months)	Median OS (months)	Refs.
**KRAS G12D inhibitor (monotherapy)**	NCT05533463 (I/II); HRS‐4642 was administered IV QW (200, 300, 400, or 500 mg), Q2W (800 or 1200 mg) or Q3W (1200 mg)	Advanced KRAS G12D‐mutant solid tumors (dose escalation)	NSCLC: 2/9 patients achieved partial response; 7/9 had stable disease	NA	NA	[113]
KRAS G12D inhibitor (combination)	NCT06520488 (I/II); HRS‐4642 + gemcitabine + nab‐paclitaxel	Advanced KRAS G12D‐mutant PDAC	63.3	NA	NA	[114]
	NCT05382559 (I); Setidegrasib vs. Setidegrasib + mFOLFIRINOX	Advanced or treatment‐naïve metastatic PDAC and NSCLC with KRAS G12D mutation	NSCLC: 36; PDAC: 24	NSCLC: 8.3; PDAC: 3.0	NSCLC: NA; PDAC: 10.3	[115]

Abbreviations: CRC, colorectal cancer; IV, intravenous; mFOLFIRINOX, modified folinic acid, fluorouracil, irinotecan, and oxaliplatin; NA, not available; NSCLC, non‐small cell lung cancer; ORR, objective response rate; OS, overall survival; PDAC, pancreatic ductal adenocarcinoma; PFS, progression‐free survival; QW, once weekly; Q2W, every 2 weeks; Q3W, every 3 weeks.

#### 3.3.2 Future Clinical Perspectives

The clinical translation of KRAS G12D inhibitors may represent the next major step in KRAS‐targeted therapy for CRC, but several key questions remain unresolved. First, it is not yet clear whether G12D inhibition alone will be sufficient to generate clinically meaningful and durable responses in human CRC. Although preclinical models have shown impressive antitumor activity, the complexity of human tumors, including intratumoral heterogeneity, adaptive signaling, and microenvironmental buffering, may substantially attenuate these effects in the clinic.

Second, lessons from KRAS G12C strongly suggest that combination therapy may again be necessary. Because both G12C and G12D ultimately drive tumor growth through MAPK pathway signaling, it is plausible that G12D inhibition in CRC may also trigger adaptive EGFR feedback or other bypass circuitry [111, 112, 116]. If so, dual‐targeting strategies incorporating EGFR antibodies, SHP2 inhibitors, or downstream pathway inhibitors may be required to maximize benefit. This possibility is especially important in CRC, where lineage‐specific signaling rewiring has already proven to be a major determinant of therapeutic response.

Third, the optimal integration of G12D inhibitors with existing CRC treatment backbones remains undefined. Rational future directions include EGFR‐based cotargeting and other pathway‐directed combinations, while integration with chemotherapy or immunotherapy remains an area for further investigation [117]. Whether G12D inhibitors can ultimately reproduce or exceed the translational success of G12C‐directed strategies will depend not only on their biochemical potency, but also on how effectively they are deployed within the broader biologic context of CRC. In this sense, G12D is not simply the “next KRAS target”; it is the next major test of whether context‐informed KRAS therapy can be generalized beyond the first success of G12C.

### 3.4 Targeting Explorations for G13D and Other Mutation Subtypes

#### 3.4.1 G13D: Structural Characteristics and Therapeutic Controversy

KRAS G13D is the third most common KRAS mutation subtype, accounting for approximately 10%–20% of KRAS‐mutant CRC cases [118, 119]. Biologically, G13D differs from the more common G12 substitutions in ways that may have therapeutic implications. Its distinct conformational and catalytic properties suggest that it should not be viewed simply as another generic KRAS‐mutant state, but rather as a subtype with partially divergent signaling behavior. This distinction is particularly relevant in CRC, where subtle differences in upstream signaling dependence may translate into meaningful differences in therapeutic response.

Among all KRAS subtypes in CRC, G13D has generated the most persistent controversy regarding sensitivity to anti‐EGFR therapy. Early clinical observations, including analyses from the CRYSTAL study, suggested that patients with G13D‐mutant tumors might derive greater benefit from cetuximab than patients harboring other KRAS mutations, raising the possibility that G13D could retain partial sensitivity to EGFR blockade [100, 120, 121, 122]. However, subsequent retrospective analyses and later datasets failed to confirm a consistent benefit, and some studies instead supported resistance to panitumumab or a lack of meaningful activity overall [123]. These conflicting results likely reflect heterogeneity in trial design, patient selection, and the historical tendency to group KRAS mutations together rather than analyzing subtype‐specific biology.

More recent mechanistic work suggests that the therapeutic behavior of G13D may be shaped not only by the KRAS allele itself, but also by co‐mutation context. In particular, NF1 status has been proposed as a potential determinant of EGFR antibody sensitivity in G13D‐mutant tumors, with loss of neurofibromin potentially promoting resistance while intact NF1 may preserve some degree of upstream dependence [124]. If confirmed, this would support a more nuanced interpretation of G13D in CRC: not as a uniformly EGFR‐sensitive subtype, but as a biologically distinct subset in which response may depend on accompanying regulatory circuitry.

Direct inhibition of G13D remains challenging. Because the mutation lies in the P‐loop and lacks the structural features that made G12C druggable, the pocket‐based covalent inhibition paradigm cannot be directly translated to G13D [125]. Nonetheless, recent structural studies have begun to identify potentially druggable conformational states, raising the possibility of future monoclonal antibody‐based or reversible small‐molecule approaches. At present, however, G13D remains more biologically interesting than therapeutically solved. The main clinical message is that G13D should be regarded as a distinct CRC subtype with unresolved but plausible subtype‐specific therapeutic behavior, rather than being simplistically classified as either fully anti‐EGFR sensitive or fully equivalent to all other KRAS mutations.

#### 3.4.2 Targeting Strategies for Rare Mutations (G12V, Q61H, etc.)

Beyond G12C, G12D, and G13D, several additional RAS mutation subtypes contribute meaningfully to the molecular heterogeneity of CRC. Among these, KRAS G12V is particularly important because it is the second most common KRAS mutation subtype in CRC, accounting for approximately 20%–30% of cases [126, 127]. Mechanistically, G12V primarily promotes oncogenic signaling by impairing GAP‐mediated GTP hydrolysis, which distinguishes it from both G12C and Q61‐driven activation states [108, 128]. Despite its prevalence, G12V currently lacks a clinically mature allele‐specific inhibitor, and therapeutic strategies therefore remain largely dependent on pan‐KRAS approaches or broader pathway‐directed combinations.

Other rare RAS alterations, including NRAS Q61 mutations such as Q61H, Q61L, and Q61R, occur less frequently in CRC but may be associated with more aggressive tumor biology [127]. Although their prognostic impact may differ somewhat from that of common KRAS mutations, they remain clinically unfavorable relative to RAS wild‐type disease [129, 130, 131]. A recent preclinical study demonstrated that NRAS/KRAS Q61X mutant tumor cells, including those harboring Q61H and Q61R substitutions, sustain downstream pathway activation through interaction with Leucine Rich Repeat Scaffold Protein (SHOC2), thereby promoting tumor growth and drug resistance. Notably, disruption of the Q61X–SHOC2 complex was shown to suppress this oncogenic signaling process [132]. These findings provide important proof of concept from both structural biology and medicinal chemistry for therapeutic targeting of Q61X‐mutant RAS. However, substantial challenges remain before such strategies can be translated into effective clinical therapies. Mechanistically, these mutations arise from profound disruption of the GTPase catalytic machinery, and no subtype‐specific targeted therapies are currently available. Therefore, current therapeutic exploration in this setting has largely focused on broader RAS‐targeting approaches and synthetic lethal strategies rather than on mutation‐specific drug development alone.

In this context, pan‐KRAS and pathway‐level strategies are particularly relevant. Agents such as SOS1 inhibitors and pan‐KRAS inhibitors may provide a broader therapeutic framework for subtypes that are clinically meaningful but chemically less tractable [108]. This is especially important in CRC, where the long tail of KRAS and NRAS variants collectively represents a substantial patient population despite the relative rarity of each individual allele. Thus, the treatment landscape for rare RAS subtypes in CRC is unlikely to be shaped by a separate drug for each mutation; instead, it will more likely depend on broader strategies capable of covering multiple biologically active but individually uncommon alleles.

### 3.5 Resistance Mechanisms in KRAS‐Targeted Therapy for Colorectal Cancer

Although combining KRAS G12C inhibitors with EGFR antibodies has established a meaningful therapeutic strategy in CRC, acquired resistance remains a major limitation. Some resistance mechanisms overlap with those observed in NSCLC, including secondary KRAS alterations and MAPK pathway reactivation (Figure 3). However, the weighting of these mechanisms differs substantially in CRC because of its pronounced dependence on upstream EGFR signaling and its distinct intestinal tumor ecology. In CRC, resistance is therefore best understood not as a generic extension of KRAS inhibitor resistance in NSCLC, but as a lineage‐specific adaptive process shaped by epithelial signaling context.

A major feature of CRC resistance biology is the contribution of the tumor microenvironment. Cancer‐associated fibroblasts and other stromal components can provide compensatory survival signals through factors such as hepatocyte growth factor, thereby activating MET signaling and promoting tumor‐cell survival and invasive behavior [133]. At the same time, the marked intratumoral heterogeneity of CRC facilitates the outgrowth of pre‐existing resistant subclones under treatment pressure, including those harboring PIK3CA alterations, PTEN loss, or epithelial–mesenchymal transition‐associated programs [134, 135]. These features help explain why even initially effective dual‐targeting approaches may fail over time.

CRC may also have resistance determinants that are less prominent in other KRAS‐driven malignancies. Because the colorectum exists in close interaction with the intestinal microbiota, microbial metabolites may influence drug sensitivity either indirectly through epigenetic remodeling or directly through effects on tumor metabolism and treatment response [136, 137, 138]. Although this area remains early, it highlights an important conceptual point: in CRC, resistance may arise not only from tumor‐intrinsic signaling rewiring, but also from microenvironmental and host‐associated factors that are specific to the intestinal setting.

Taken together, resistance to KRAS‐targeted therapy in CRC is shaped by both shared oncogenic escape routes and uniquely colorectal determinants, especially EGFR dependence, stromal signaling, clonal heterogeneity, and intestinal microenvironmental influences. This has direct therapeutic implications. In CRC, rational combination therapy is not merely an optional intensification strategy; it is likely required from the outset in many patients because durable control depends on suppressing the adaptive network surrounding KRAS rather than on KRAS inhibition alone.

## 4 Pancreatic Cancer: Multi‐Subtype Targeting Under a G12D‐Dominant Landscape

PDAC represents a KRAS G12D‐dominant but therapeutically high‐barrier disease setting. This section first reviews the distinctive KRAS mutation spectrum of PDAC, in which G12D predominates, G12V remains common, G12R is selectively enriched, and G12C is rare. We then discuss the progress and remaining challenges of G12D‐directed drug development, followed by the limited therapeutic tractability of G12V and G12R. Pan‐KRAS inhibitors are evaluated as a potential means of expanding subtype coverage, while their therapeutic window, mutant selectivity, and uneven activity across alleles are critically considered. Finally, we examine the dense desmoplastic stroma, limited vascularity, immunosuppressive microenvironment, autophagy dependence, and metabolic plasticity that restrict treatment efficacy in PDAC. These features indicate that successful KRAS‐directed therapy in pancreatic cancer will require not only effective target engagement but also therapeutic reshaping of the surrounding tumor context.

### 4.1 Molecular Epidemiology of KRAS Mutations in Pancreatic Cancer

KRAS is the dominant driver alteration in PDAC, occurring in approximately 85%–90% of cases and shaping both tumor biology and therapeutic vulnerability [139]. Compared with other solid tumors, the mutational spectrum of KRAS in PDAC is relatively concentrated, with G12D as the predominant subtype, followed by G12V, whereas G12C is rare and of limited clinical relevance in this disease setting [140, 141]. Notably, G12R occurs at a substantially higher frequency in PDAC than in most other malignancies, underscoring the distinct lineage‐specific biology of pancreatic cancer [142]. Taken together, this distribution pattern indicates that KRAS‐targeted therapy in PDAC cannot be modeled simply on the NSCLC G12C paradigm, but instead requires a multi‐subtype strategy centered on G12D while also accounting for G12V and the PDAC‐enriched G12R subtype [143].

### 4.2 G12D: The Core Therapeutic Priority in Pancreatic Cancer

Among KRAS‐mutant pancreatic cancers, G12D represents the most important therapeutic priority. As in CRC, the main obstacle is structural: unlike G12C, G12D does not provide a cysteine residue for covalent engagement, forcing drug development toward highly optimized non‐covalent approaches. This challenge is particularly relevant in PDAC because the high prevalence of G12D intersects with the dire prognosis of the disease, making effective G12D inhibition a major unmet clinical need [144, 145]. Encouragingly, the G12D therapeutic pipeline has advanced substantially in recent years. MRTX1133 provided early proof of concept that potent and selective G12D inhibition is achievable and can induce marked antitumor effects in preclinical pancreatic ductal adenocarcinoma models [146]. Additional observations suggesting immune microenvironmental remodeling after G12D inhibition have further strengthened the rationale for combination strategies, particularly with immunotherapy or other context‐directed approaches [147, 148]. Other agents, including HRS‐4642 and RMC‐9805, have expanded the early clinical and translational landscape (Table 3), while additional candidates remain in preclinical development [15]. Together, these advances indicate that KRAS G12D has moved from a historically “undruggable” concept to an active area of therapeutic development, although its ultimate clinical value in PDAC remains to be established.

### 4.3 G12V And G12R: Clinically Relevant but Less Tractable Subtypes

#### 4.3.1 G12V: A Common but Still Under‐Served Subtype

G12V is the second most common KRAS mutation subtype in PDAC and therefore cannot be considered a marginal molecular subgroup. However, like G12D, it lacks a cysteine residue that would enable covalent targeting, and no mature allele‐specific inhibitors are currently available for routine clinical use [149, 150]. As a result, patients with G12V‐mutant PDAC are presently more likely to benefit from broader pan‐KRAS strategies or pathway‐based combinations than from subtype‐specific inhibition. This limitation highlights an important translational reality in PDAC: clinical need is not always aligned with current druggability.

#### 4.3.2 G12R: A Pancreatic Cancer‐Enriched Subtype With Distinct Biology

G12R deserves particular attention because it is enriched in PDAC relative to most other solid tumors and may reflect a distinct pattern of KRAS signaling dependency in pancreatic cancer [7, 151]. Although its biochemical behavior is not yet fully resolved, available studies suggest that G12R may differ from G12D and G12V in its interaction with upstream regulators and downstream effectors [152, 153]. At present, no G12R‐specific inhibitors are available, and therapeutic strategies remain largely dependent on pan‐KRAS approaches. However, preliminary evidence suggests that some pan‐KRAS platforms may be less active against G12R than against other KRAS subtypes, raising the possibility that PDAC patients with G12R mutations could remain relatively under‐covered even within broader KRAS‐directed approaches. Accordingly, future work should not only expand pan‐KRAS coverage, but also consider whether G12R requires its own context‐specific therapeutic logic.

### 4.4 Pan‐KRAS Inhibitors: Broad Opportunity, but Not a Universal Solution

#### 4.4.1 Development Progress

Pan‐KRAS inhibition is especially attractive in PDAC because this disease contains multiple clinically relevant KRAS subtypes, whereas currently approved or near‐clinical agents remain heavily skewed toward individual alleles. RMC‐6236 is among the most advanced agents in this category and has helped establish the feasibility of targeting multiple KRAS mutants through a tri‐complex mechanism [15, 83]. Additional strategies, including pan‐KRAS degraders and indirect inhibitors such as SOS1 inhibitors, further broaden the field and illustrate that pan‐KRAS therapy is now a legitimate translational direction rather than a purely theoretical one (Table 2) [141, 154, 155, 156, 157]. Daraxonrasib has now advanced beyond early clinical development, with the phase III RASolute 302 trial demonstrating significant survival benefit over standard chemotherapy in previously treated metastatic PDAC. This milestone provides clinical proof of concept for multi‐selective RAS inhibition and substantially strengthens the translational relevance of pan‐RAS strategies in pancreatic cancer.

#### 4.4.2 Advantages and Challenges

The conceptual advantages of pan‐KRAS strategies are clear: they may cover multiple mutant subtypes simultaneously, reduce dependence on perfect allele resolution in limited tissue samples, and potentially suppress the emergence of resistant subclones by targeting a broader KRAS‐mutant space. These features are particularly appealing in PDAC, where G12D, G12V, and G12R coexist at meaningful frequencies [158]. At the same time, pan‐KRAS inhibition raises substantial translational challenges (Table 2). The most important is therapeutic window: excessive inhibition of wild‐type KRAS could produce on‐target toxicity in normal tissues, especially in the gastrointestinal tract and other rapidly renewing compartments. In addition, broader molecular coverage does not necessarily imply equivalent inhibition across all KRAS variants. Some GDP‐state‐selective pan‐KRAS inhibitors show reduced activity against KRAS G12R, highlighting allele‐dependent differences even within broadly targeted KRAS platforms [159, 160]. Finally, as these agents move into combination regimens, additive toxicity will become a central issue. Thus, pan‐KRAS inhibitors should be viewed as an important strategic expansion of the field, but not yet as a definitive one‐size‐fits‐all solution [161].

### 4.5 Unique Challenges and Combination Strategies in PDAC

Even if direct KRAS inhibition becomes technically feasible in PDAC, the pancreatic tumor context remains a major constraint on efficacy. PDAC is characterized by a dense stromal architecture, low vascularity, abundant cancer‐associated fibroblasts, and a profoundly immunosuppressive microenvironment. These features impair drug delivery, buffer signaling stress, and protect tumor cells from therapy‐induced killing [162]. In parallel, pancreatic cancer cells are highly adaptable metabolically and can rewire autophagy, glutamine metabolism, and lipid metabolism in response to treatment pressure. This combination of physical barrier and metabolic plasticity helps explain why KRAS inhibition in PDAC may be less effective than expected from genotype alone [163, 164]. Accordingly, combination therapy is likely to be particularly important in pancreatic cancer. Chemotherapy‐based combinations may provide immediate cytoreduction, whereas stromal and microenvironment‐directed strategies, including FAK inhibition, may help overcome contextual barriers that limit therapeutic efficacy and durability (Figure 3) [165]. The key issue moving forward is not simply whether combinations can increase activity, but which biologic bottleneck is dominant in a given PDAC setting and how to combine therapies without compromising tolerability. In PDAC more than in any other KRAS‐driven malignancy, the success of KRAS‐targeted therapy will depend on whether tumor context can be therapeutically reshaped.

## 5 KRAS‐Targeted Therapy in Other Solid Tumors

Beyond NSCLC, CRC, and PDAC, KRAS‐targeted therapy is being explored across a heterogeneous group of less common solid tumors, but the available evidence remains uneven and frequently limited by small molecularly defined populations. This section reviews the emerging therapeutic landscape in cholangiocarcinoma, endometrial cancer, gastric cancer, and other rare malignancies. Particular attention is given to how anatomic subtype, KRAS allele distribution, co‐occurring pathway alterations, and tumor‐specific biology influence therapeutic vulnerability. In cholangiocarcinoma and gastric cancer, current evidence is derived mainly from small cohorts and basket trials, whereas the frequent coexistence of KRAS and PI3K‐pathway alterations in endometrial cancer provides a rationale for biologically informed cotargeting. In rarer tumors, the predominance of non‐G12C variants and the limited feasibility of conventional disease‐specific trials highlight the importance of pan‐KRAS approaches, basket‐trial designs, real‐world evidence, and collaborative molecular registries. Collectively, these settings demonstrate that mutation frequency does not necessarily predict therapeutic tractability or the strength of clinical evidence.

### 5.1 Cholangiocarcinoma

CCA illustrates the intermediate zone between common KRAS‐driven cancers and truly rare molecular settings. KRAS mutations occur in a meaningful minority of CCA cases, with subtype distribution varying by anatomic location [166, 167]. For patients with G12C‐mutant CCA, case reports and small case series of sotorasib and adagrasib have been reported. A pooled analysis incorporating these data showed an ORR of approximately 41.7% with G12C inhibitors [48], with a median progression‐free survival of 8.6 months. Available evidence suggests that G12C‐directed therapy can produce clinical activity in a subset of patients, but the magnitude of benefit appears lower than in NSCLC and the evidence base remains limited to small cohorts, pooled analyses, or basket‐trial experience [166]. As a result, KRAS‐targeted therapy in CCA remains promising but not yet practice‐defining. For non‐G12C subtypes such as G12D and G12V, clinical options remain largely investigational and are more likely to rely on pan‐KRAS platforms or basket‐trial enrollment [154]. Given the rarity and anatomic heterogeneity of CCA, future progress will probably depend on multi‐institutional collaboration, disease‐agnostic enrollment strategies, and careful interpretation of real‐world evidence rather than on conventional large registrational studies alone [163].

### 5.2 Endometrial Cancer

KRAS‐mutant endometrial cancer (EC) presents a different therapeutic logic because KRAS alterations frequently coexist with PI3K pathway abnormalities [168]. This co‐mutation pattern is important, because it may both influence prognosis and shape therapeutic vulnerability. At present, direct clinical evidence for KRAS‐targeted therapy in EC remains sparse and is derived mainly from small basket‐trial subsets, making any efficacy conclusion provisional. The number of G12C‐mutant EC patients enrolled in the CodeBreaK and KRYSTAL basket trials is extremely limited (typically in the single digits), with objective response rates of approximately 30%–40% [169]. Still, the available signals suggest that KRAS‐directed approaches may be biologically relevant in at least a subset of cases. Preclinical studies of multi‐selective RAS inhibitors such as RMC‐6236 have demonstrated broad antitumor activity across KRAS G12X‐driven tumor models; however, endometrial cancer‐specific clinical evidence remains immature [154]. What makes EC especially interesting is not the maturity of current KRAS‐targeted evidence, but the plausibility of rational cotargeting. Because KRAS and PI3K pathway alterations commonly coexist, dual‐pathway inhibition may be more compelling here than in several other tumor types [170]. Thus, EC may ultimately become an example of a disease in which the value of KRAS‐directed therapy lies less in single‐agent activity and more in biologically informed combinations. Preclinical studies across multiple tumor models suggest that combining KRAS inhibitors with PI3K inhibitors (such as alpelisib) or mTOR inhibitors (such as everolimus) may produce synergistic effects [171, 172]. In addition, KRAS mutation status may have prognostic value—multiple retrospective studies have shown that patients with KRAS‐mutant EC have shorter progression‐free survival and overall survival than those with KRAS wild‐type tumors [173, 174].

### 5.3 Gastric Cancer

Gastric cancer (GC) remains an exploratory setting for KRAS‐targeted therapy. The incidence of KRAS mutations in GC is approximately 5%–10%, markedly lower than that in colorectal or pancreatic cancer, and the subtype distribution is relatively heterogeneous, with G12D, G12V, and G13D all being reported [10]. In addition, the prevalence and spectrum of KRAS mutations may vary according to geographic region and histologic subtype. For example, several studies have suggested that KRAS mutations may be less frequent in diffuse‐type GC than in intestinal‐type GC, although the available datasets remain too limited to support firm conclusions [175]. These features indicate that KRAS‐mutant GC is not only rare, but also biologically heterogeneous.

Direct clinical evidence for KRAS‐targeted therapy in GC remains sparse. To date, no prospective clinical trial has specifically established the efficacy of KRAS‐targeted therapy in gastric cancer. Although early basket studies of KRAS G12C inhibitors have demonstrated activity across several non‐NSCLC and non‐CRC solid tumors, gastric cancer‐specific efficacy has not been adequately characterized, as gastric cancer representation in these studies has been extremely limited or not separately reported [48, 176]. Thus, unlike NSCLC and, to a lesser extent, CRC, GC still lies at a very early stage of translational development in the KRAS field.

For non‐G12C subtypes such as G12D and G12V, pan‐KRAS strategies may offer broader theoretical relevance, but disease‐specific evidence remains minimal. Preclinical studies of RMC‐6236 and related approaches have shown activity in selected GC cell lines and xenograft models, although the magnitude of response appears less pronounced than that seen in pancreatic or colorectal models [108, 154]. This attenuated activity may reflect differences in signaling dependency, co‐occurring genomic alterations, or the gastric tumor microenvironment [176]. These observations are important because they suggest that the limited efficacy of KRAS‐directed therapy in GC is unlikely to be explained by mutation frequency alone; rather, it may also reflect a distinct biologic context in which KRAS is not the sole dominant driver of therapeutic response.

Moving forward, progress in KRAS‐mutant GC will likely depend on two complementary strategies. First, larger basket‐style studies and real‐world datasets will be required to accumulate sufficient patient numbers for meaningful clinical interpretation [175]. Second, more granular molecular characterization will be essential, because GC is a highly heterogeneous disease in which immune infiltration patterns, stromal features, and co‐mutation landscapes vary substantially across molecular subtypes. In particular, whether distinct GC subgroups—such as Epstein–Barr virus‐positive or microsatellite instability‐high tumors—modulate sensitivity to KRAS‐directed therapy remains unknown and warrants explicit investigation in future studies. In this sense, KRAS‐targeted therapy in GC is best viewed not as a mature therapeutic option, but as a biologically plausible yet still weakly evidenced frontier.

### 5.4 Other Rare Cancer Types and Basket Trial Evidence

Beyond the more commonly discussed tumor types, KRAS mutations are also found in a range of rare solid tumors, including small bowel cancer (SBC), appendiceal cancer, cervical cancer (CC), and ovarian cancer (OC), with mutation frequencies and subtype distributions varying substantially according to tumor type and histologic subtype [177, 178]. In these malignancies, the low absolute number of patients and the wide variation in histology make conventional disease‐specific drug development particularly difficult. As a result, most available clinical evidence for KRAS‐targeted therapy derives from basket trials, pooled subgroup analyses, or isolated case‐level observations rather than from tumor‐specific prospective studies.

Current evidence suggests that the activity of KRAS G12C inhibitors in rare tumors is highly variable and, in many settings, difficult to interpret because of very small sample sizes. Pooled analyses from basket trials indicate that G12C inhibitors can produce modest antitumor activity in selected rare malignancies such as cholangiocarcinoma and endometrial cancer, but the corresponding evidence in other rare KRAS‐mutant malignancies remains extremely limited [48, 177, 178]. Consequently, the existing data are not sufficient to define either a tumor‐specific standard or a reliable estimate of response magnitude for most rare KRAS‐mutant cancers.

An additional complication is that the subtype distribution of KRAS mutations in rare tumors often differs from that observed in NSCLC. In ovarian and cervical cancers, for example, G12D and G12V appear to predominate, whereas G12C is exceptionally uncommon. This has practical therapeutic implications: even where direct G12C inhibition is clinically validated, the eligible population in these rare tumor types may remain intrinsically very small. Therefore, the future value of KRAS‐targeted therapy in rare cancers may depend less on the expansion of G12C inhibitors and more on the development of broader strategies, including pan‐KRAS inhibitors and non‐G12C‐directed approaches.

As the development of pan‐KRAS inhibitors continues to accelerate, the outlook for patients with rare KRAS‐mutant solid tumors may gradually improve (Table 2). However, because randomized controlled trials are often not feasible in ultra‐rare molecular subsets, future decision‐making will likely rely increasingly on real‐world evidence, long‐term follow‐up from basket studies, and harmonized cross‐institutional registries. In this setting, evidence generation itself becomes part of the therapeutic challenge. For rare KRAS‐mutant tumors, the question is not only whether a drug can work, but also how clinically meaningful evidence can be built in populations too small for traditional trial paradigms.

## 6 Summary and Perspectives

KRAS‐mutant malignancies were long regarded as one of the archetypal “undruggable” territories in oncology. For decades, the smooth surface of KRAS, its exceptionally high affinity for GTP, and the lack of an obvious drug‐binding pocket collectively prevented the development of effective direct inhibitors. This landscape changed fundamentally with the clinical success of covalent KRAS G12C inhibitors, beginning with the approval of sotorasib and followed by the broader maturation of the G12C field. These breakthroughs established proof of principle that direct KRAS inhibition is clinically achievable and transformed KRAS from a symbolic challenge into a rapidly evolving therapeutic class.

However, the most important lesson from the past several years is not simply that KRAS can be targeted, but that the consequences of KRAS inhibition are profoundly shaped by tumor context. The same KRAS allele can have very different therapeutic implications across tumor types because lineage‐specific signaling dependency, feedback circuitry, co‐mutation architecture, stromal barriers, and pharmacologic exposure all influence whether target engagement translates into durable clinical benefit. This context dependence explains why KRAS G12C inhibitors have established clear, though still incomplete, utility in NSCLC, whereas in CRC meaningful benefit emerged only after cotargeting EGFR‐mediated adaptive feedback [179, 180]. In PDAC, by contrast, the challenge is even broader: beyond the chemistry of G12D inhibition, therapeutic efficacy is further constrained by dense stroma, metabolic plasticity, and a profoundly immunosuppressive microenvironment (Figure 3) [145, 181, 182]. Thus, KRAS‐targeted therapy has not followed a single universal trajectory; instead, it has evolved along tumor‐type‐specific paths.

### 6.1 Decoding the “Same Mutation, Different Outcomes” Paradox

A central unresolved question is why an identical KRAS mutation can produce markedly different therapeutic outcomes across tumor types. The contrasting activity of KRAS G12C inhibitors in NSCLC and CRC provides one of the clearest examples of tumor‐lineage‐dependent therapeutic response [183]. Although the molecular target is identical, lineage‐specific signaling dependencies determine whether direct target engagement results in durable pathway suppression [41]. CRC is characterized by pronounced EGFR‐mediated adaptive feedback, whereas NSCLC displays a broader spectrum of resistance mechanisms, including receptor tyrosine kinase bypass, secondary KRAS alterations, mitogen‐activated protein kinase pathway rebound, and phenotypic plasticity. In PDAC, stromal and metabolic barriers further constrain drug delivery and tumor‐cell dependence on KRAS signaling [162, 164, 181].

These observations suggest that KRAS mutation status alone is insufficient as a predictive biomarker [184]. Future patient stratification should integrate the specific KRAS allele with co‐occurring genomic alterations, baseline receptor tyrosine kinase activity, tumor lineage, immune context, and pharmacologic features such as central nervous system exposure [76]. For example, STK11/LKB1 co‐mutation defines a distinct immune context in KRAS‐mutant lung adenocarcinoma and has been associated with primary resistance to PD‐1 blockade [185]. More recent KRYSTAL‐1 analyses further showed that STK11 status stratifies outcomes among patients receiving adagrasib, supporting the clinical relevance of co‐mutation and transcriptional context [186]. Longitudinal circulating tumor DNA analysis, serial tissue sampling, and integrated genomic, transcriptomic, proteomic, and spatial profiling may help identify whether an individual tumor is dominated by on‐target resistance, bypass signaling, lineage reprogramming, or microenvironmental protection. Such approaches could enable treatment selection to shift from static mutation matching toward dynamic, context‐informed therapeutic decision‐making [41, 73].

A related challenge is determining whether the biologic context remains stable during treatment. Acquired resistance may not simply introduce an additional mutation; it may change the signaling state or histologic identity of the tumor. Adeno‐to‐squamous transition, epithelial‐to‐mesenchymal transition, and other forms of phenotypic plasticity can reduce the original dependence on the signaling context in which KRAS inhibition was effective. In CRC, paired biopsy analyses have further demonstrated that subclonal genetic alterations can coexist with heterogeneous mesenchymal, yes‐associated protein (YAP), and fetal‐like transcriptional states during KRAS‐directed therapy [187]. Therefore, future biomarker strategies must not only predict initial sensitivity but also monitor evolving tumor states during treatment.

### 6.2 Closing the Therapeutic Coverage Gap Between G12C and Non‐G12C Mutations

A second major priority is closing the substantial therapeutic gap between KRAS G12C and the more prevalent but less tractable non‐G12C variants. The success of covalent G12C inhibitors was enabled by the cysteine residue introduced at codon 12, whereas G12D, G12V, G12R, G13D, and Q61 variants lack an equivalent chemical handle [21, 108, 146]. Consequently, drug development for these mutations has required alternative strategies, including non‐covalent inhibitors, active‐state tri‐complex inhibitors, pan‐KRAS platforms, RNA‐based therapies, and targeted protein degradation [188, 189, 190]. Next‐generation G12C inhibitors remain important because current agents do not fully address intracranial disease, active‐state KRAS, or the broad spectrum of secondary resistance mutations. New compounds are therefore being developed to improve potency, central nervous system penetration, pharmacokinetic properties, and activity against resistance‐associated conformations [43, 76, 191]. However, molecular optimization within G12C alone cannot address the majority of patients with KRAS‐mutant gastrointestinal cancers.

KRAS G12D represents the most immediate non‐G12C priority because of its high prevalence in PDAC and CRC. Early clinical development has begun to establish the feasibility of selective G12D inhibition, but important questions remain regarding the durability of monotherapy, tumor‐type‐specific feedback, and the need for combination therapy [88, 146, 192, 193]. G12V and G12R remain even less well covered, despite the clinical relevance of G12V in both pancreatic and colorectal cancers and the selective enrichment of G12R in PDAC [108, 128, 194]. G13D and Q61 alterations present additional structural and biologic challenges and may require mutation‐specific or synthetic‐lethal approaches rather than simple extension of the G12C paradigm. Pan‐KRAS and multi‐selective RAS inhibitors may provide a broader solution by covering several recurrent alleles simultaneously [149, 150]. Their development is particularly attractive in PDAC and rare tumors, where multiple non‐G12C variants coexist and conventional allele‐specific drug development may be impractical. Nevertheless, broad coverage introduces a different set of challenges, including inhibition of wild‐type KRAS, toxicity in normal tissues, uneven potency across mutant alleles, and the possibility that nominally pan‐KRAS agents may still leave clinically important variants insufficiently suppressed [188]. Future development should therefore define allele‐specific pharmacodynamic thresholds and therapeutic windows rather than assuming equivalent activity across all KRAS mutations.

### 6.3 From Monotherapy to Resistance‐Informed Combination Therapy

A third major direction is the transition from empirically selected monotherapy to biologically informed combination therapy. The efficacy ceiling of current KRAS inhibitors reflects incomplete pathway suppression, rapid adaptive feedback, clonal heterogeneity, and survival support from parallel signaling or the tumor microenvironment [71]. Combination therapy should therefore not be viewed simply as treatment intensification, but as a strategy for suppressing the dominant escape mechanism in a specific disease context.

CRC provides the most mature example of this principle. Direct KRAS G12C inhibition rapidly induces EGFR‐dependent pathway reactivation, and simultaneous EGFR blockade has therefore become an essential component of effective treatment in this setting. In NSCLC, the resistance landscape is more heterogeneous. Potential combinations include inhibition of SHP2 or SOS1 to reduce upstream nucleotide exchange, MEK or ERK inhibition to suppress downstream pathway rebound, and selected PI3K‐AKT‐mTOR‐directed strategies to address parallel survival signaling [41, 42, 73]. FAK/SRC signaling has also recently emerged as an adaptive resistance hub capable of reactivating RAS/MAPK and AKT signaling following KRAS G12C inhibition, providing an additional rationale for cotargeting strategies [195]. Chemo‐/immunotherapy combinations may further enhance tumor‐cell killing or immune recognition, but their clinical value must be balanced against hepatotoxicity, gastrointestinal toxicity, pulmonary toxicity, and the potential loss of dose intensity.

In PDAC, the logic of combination therapy must extend beyond intracellular signaling. Dense stroma, cancer‐associated fibroblasts, autophagy dependence, altered nutrient utilization, and immune suppression can all buffer the consequences of KRAS inhibition [147, 148]. Accordingly, combinations with chemotherapy, stromal modulators, autophagy inhibitors, metabolic interventions, or immune‐remodeling strategies may be required to convert molecular target engagement into durable tumor regression.

The central clinical question is therefore not whether combination therapy is needed, but which combination should be used, when it should be introduced, and how it should be sequenced. Upfront combinations may be appropriate when a dominant adaptive mechanism is predictable, as illustrated by EGFR feedback in CRC [53]. By contrast, sequential or biomarker‐triggered treatment may be preferable when resistance is heterogeneous or toxicity limits simultaneous administration. Future trials should incorporate molecularly defined cohorts, longitudinal resistance monitoring, pharmacodynamic assessment, and prespecified sequencing strategies rather than evaluating combinations solely through conventional response‐rate comparisons.

### 6.4 Modality Diversification and Biomarker‐Guided Therapeutic Development

Small‐molecule inhibitors will remain central to KRAS‐directed therapy, but future progress is unlikely to depend on occupancy‐driven inhibition alone. Targeted protein degraders may eliminate mutant KRAS rather than merely occupying a binding pocket, potentially producing more sustained pathway suppression and overcoming selected resistance mechanisms [94, 95, 196]. Immune‐based approaches, including mutation‐specific T‐cell receptor therapies, neoepitope‐directed strategies, and bispecific immune‐engagement platforms, may enable recognition of intracellular KRAS‐derived antigens that cannot be directly accessed by conventional antibodies [96, 97].

Antibody–drug conjugates (ADCs) represent a complementary rather than directly KRAS‐targeted strategy. Most currently available ADCs do not recognize KRAS itself; instead, they exploit tumor‐associated surface antigens or context‐dependent vulnerabilities that coexist with KRAS mutations to deliver cytotoxic payloads (Table 4). Their potential value may therefore lie in treating KRAS‐mutant tumors that remain dependent on surface targets such as TROP2, HER2, or HER3, or in overcoming resistance that is no longer driven exclusively by KRAS signaling [197, 198, 199, 200]. However, current evidence is largely derived from small molecular subgroups, exploratory analyses, or studies in which KRAS mutation was a coexisting feature rather than the direct therapeutic target. ADCs should therefore be presented as an emerging context‐directed platform rather than an established component of KRAS‐specific therapy.

**TABLE 4:** Selected clinical activity of antibody‐drug conjugates in KRAS‐mutant or KRAS‐associated solid tumors.

Therapeutic strategy	Trial (phase); treatment arms	Disease setting (n)	ORR (%)	Median PFS (months)	Median OS (months)	Refs.
**TROP2‐targeted ADC**	TROPION‐Lung01 (III); Dato‐DXd vs. docetaxel	Previously treated advanced or metastatic NSCLC (*n* = 604); efficacy data are from the overall trial population and are not KRAS‐specific	Dato‐DXd: 26.4; docetaxel: 12.8	Dato‐DXd: 4.4; docetaxel: 3.7 (HR 0.75)	Dato‐DXd: 12.9; docetaxel: 11.8 (HR 0.94)	[198]
	EVOKE‐01 (III); sacituzumab govitecan vs. docetaxel	Previously treated advanced NSCLC; exploratory KRAS‐mutant subgroup analysis	NA	NA	10.0 vs. 9.3 (HR 0.96)	[197, 201]
**HER2‐targeted ADC**	DESTINY‐Lung01 (II); trastuzumab deruxtecan (6.4 mg/kg)	HER2‐mutant, KRAS co‐mutation NSCLC (*n* = 17)	54.9 (overall HER2‐mutant cohort, *n* = 91)	8.2 (overall HER2‐mutant cohort)	17.8 (overall HER2‐mutant cohort)	[199]
**HER3‐targeted ADC**	TUXEDO‐3 (II); patritumab deruxtecan (5.6 mg/kg Q3W)	NSCLC with active brain metastases (*n* = 20); one intracranial responder harbored KRAS G12C	Intracranial ORR: 30	NA	NA	[200]

*Note*: For TROPION‐Lung01, DESTINY‐Lung01, and TUXEDO‐3, efficacy outcomes are derived from the overall study populations and should not be interpreted as KRAS‐specific efficacy estimates; these studies are included to illustrate the potential relevance of ADCs in KRAS‐associated therapeutic contexts.

Abbreviations: ADC, antibody–drug conjugate; HER2, human epidermal growth factor receptor 2; HER3, human epidermal growth factor receptor 3; HR, hazard ratio; NA, not available; NE, not evaluated; NSCLC, non‐small cell lung cancer; ORR, objective response rate; OS, overall survival; PFS, progression‐free survival; Q3W, every 3 weeks; TROP2, trophoblast cell‐surface antigen 2.

In parallel, improved drug‐delivery technologies may be particularly important in anatomically protected or pharmacologically difficult settings. CNS‐penetrant inhibitors are needed for patients with brain metastases, whereas delivery systems capable of improving tumor penetration may be valuable in stromal‐rich PDAC. These platform‐level innovations should be accompanied by biomarker‐guided trial design, because the effectiveness of a new modality will depend on target expression, allele context, resistance state, and tissue‐specific accessibility.

Evidence generation will also need to evolve. Large randomized trials may remain feasible for common settings such as KRAS G12C‐mutant NSCLC, but basket trials, molecular registries, and real‐world datasets will be increasingly important for G12R, G13D, Q61 alterations, and rare KRAS‐mutant tumors. Harmonized molecular annotation and standardized reporting of allele, tumor type, co‐mutation status, prior KRAS inhibitor exposure, intracranial disease, and resistance mechanisms will be essential for interpreting results across small and heterogeneous patient populations.

### 6.5 Concluding Remarks

In summary, KRAS‐targeted therapy has moved from a conceptual impossibility to a central frontier of precision oncology. The first generation of clinical breakthroughs answered whether KRAS could be directly drugged. The next phase must address a more complex set of questions: why the same mutation produces different outcomes across tissues, how therapeutic coverage can be expanded beyond G12C, and how resistance can be anticipated and suppressed through context‐matched combinations. The future of the field will therefore not be defined by a single best inhibitor or a universal treatment regimen. Durable benefit will require integration of allele‐specific pharmacology, tumor‐lineage biology, resistance monitoring, combination design, and emerging therapeutic modalities. Ultimately, progress will depend on matching the right KRAS‐directed or KRAS‐associated strategy to the right patient, tumor context, and stage of disease evolution.

## Author Contributions

**Leilei Wu** and **Jiaying Cai**: conceptualization, methodology, validation, investigation, writing – original draft. **Yimin Wu** and **Wenmei Jiang**: methodology, validation, investigation, writing – original draft. **Xing Jia, Li Yang, Jue Shen,** and **Wenjun Yang**: methodology, validation, investigation, writing – original draft. **Zixuan Fei, Yang Pan, Jiazheng Zhao,** and **Junnan Ru**: formal analysis, writing – review & editing. **Da Chen, Taobo Luo,** and **Weishan Lu**: formal analysis, writing – review & editing. **Zhiye Huang, Haihong Yu,** and **Haihan Deng**: formal analysis, writing – review & editing. **Jian Zeng**: conceptualization, supervision, formal analysis, writing – review & editing. All authors have read and approved the final manuscript.

## Funding

This work was supported by Zhejiang Provincial Medical Health Science and Technology Project (grant nos.: 2025KY695, 2023KY619, and 2024KY800) and Zhejiang Provincial Cancer Hospital Cultivation Fund (grant nos.: PY2024064 and PY2024034).

## Ethics Statement

The authors have nothing to report.

## Conflicts of Interest

The authors declare no conflicts of interest.

## Data Availability

The authors have nothing to report.
